# Understanding ethnic inequalities in cancer diagnostic intervals: a cohort study of patients presenting suspected cancer symptoms to GPs in England

**DOI:** 10.3399/BJGP.2024.0518

**Published:** 2025-03-25

**Authors:** Tanimola Martins, Liz Down, Alfred Samuels, Deepthi Lavu, William Hamilton, Gary Abel, Richard D Neal

**Affiliations:** University of Exeter Collaboration for Academic Primary Care (APEx), University of Exeter Medical School, Exeter.; University of Exeter Collaboration for Academic Primary Care (APEx), University of Exeter Medical School, Exeter.; National Institute for Health and Care Research (NIHR), Applied Research Collaboration (ARC) Southwest Peninsula (PenARC), University of Exeter, Exeter.; University of Exeter Collaboration for Academic Primary Care (APEx), University of Exeter Medical School, Exeter.; University of Exeter Collaboration for Academic Primary Care (APEx), University of Exeter Medical School, Exeter.; National Institute for Health and Care Research (NIHR), Applied Research Collaboration (ARC) Southwest Peninsula (PenARC), University of Exeter, Exeter.; University of Exeter Collaboration for Academic Primary Care (APEx), University of Exeter Medical School, Exeter.

**Keywords:** diagnostic interval, diagnostic pathway, ethnic inequalities, early detection, primary care, symptomatic cancer

## Abstract

**Background:**

UK Asian and Black patients experience longer cancer diagnostic intervals — the period between initial symptomatic presentation in primary care and cancer diagnosis.

**Aim:**

To determine whether the differences in diagnostic intervals are because of prolonged primary care, referral, or secondary care interval.

**Design and setting:**

A cohort study was undertaken of 70 971 patients with seven cancers (breast, lung, prostate, colorectal, oesophagogastric, myeloma, ovarian) diagnosed after symptom presentation in English primary care.

**Method:**

Data on symptom presentation and diagnosis were extracted from cancer registry-linked primary care and secondary care data. Primary interval was defined as the period between first primary care presentation and secondary care referral, referral interval as the period between referral and first secondary care appointment, and secondary care interval as the period between the first secondary care appointment and diagnosis. Accelerated failure time models were used to investigate ethnic differences across all four intervals.

**Results:**

Across all sites, the median diagnostic interval was 46 days, ranging from 13 days for breast cancer to 116 days for lung cancer. It was 14% longer for Black patients (adjusted time ratio [ATR] 1.14, 95% confidence interval [CI] = 1.05 to 1.25) and 13% longer for Asian patients (ATR 1.13, 95% CI = 1.03 to 1.23) compared with White patients. Site-specific analyses showed that, for myeloma, lung, prostate, and colorectal cancer, the secondary care interval was longer in Asian and Black patients, who also had a longer primary care interval in breast and colorectal cancer. There was little evidence of ethnic differences in referral interval.

**Conclusion:**

This study found evidence of ethnic differences in diagnostic intervals, with prolonged secondary care intervals for four common cancers and prolonged primary care intervals for two. Although these differences are relatively modest, they are unjustified and may indicate shortcomings in healthcare delivery that disproportionately affect ethnic minorities.

## Introduction

For some cancer types, UK Asian and Black ethnic minority groups are more likely than their White counterparts to have advanced-stage diagnosis and poorer survival.^1^^–^^3^ These ethnic groups also report suboptimal care experiences, including both primary and specialist care.^4^^–^^7^ Understanding the mechanisms underlying these disparities is critical for developing effective interventions.

Previous research on the determinants of ethnic variations in cancer outcomes has focused largely on tumour biology and patient factors, such as poorer cancer awareness, low screening uptake, and delayed medical help-seeking, all being less favourable for the minority groups.^8^^–^^15^ However, emerging evidence suggests that modifiable healthcare system-related factors could also play a role, although the underlying mechanism is not fully understood. For instance, the current author group in a recent population-based study of patients presenting symptoms of seven common cancer types in primary care,^16^ showed that British Asian and Black groups experienced longer diagnostic intervals (DIs — the period between initial symptomatic presentation in primary care and diagnosis of cancer) compared with their White counterparts.^17^

The DI incorporates three distinct periods: the primary care interval (PI), referral interval (RI), and secondary care interval (SI), all defined in the ‘Methods — Defining DIs’.^18^ None of these intervals were specifically examined in the current authors’ previous study.^17^ Understanding which of these subintervals is most relevant will help determine whether interventions to address inequalities should target primary care, secondary care, or both; although interventions applied to one may have an impact on the other. Extending the current authors’ previous work, in this study, cancer registry-linked primary care and secondary care data were used to investigate ethnic differences in PI, RI, and SI. As most ‘delays’ in cancer diagnosis are attributed to the PI,^19^^,^^20^ the authors hypothesised that much of the differences in the DI by ethnicity would also be explained by the differences in PI rather than RI or SI.

**Table:** How this fits in

Previous research by the current authors demonstrated that, when compared with White patients, Asian and Black patients in the UK experience longer diagnostic intervals for common cancers. The present study aimed to determine whether these disparities stem from delays in primary care, secondary care, or both, to identify the setting where targeted interventions may be most effective. Contrary to the authors’ hypothesis, the results indicated that, for four of the seven cancers studied, delays primarily occurred in secondary care — an area that has received comparatively less attention. Future work should focus on identifying the causes of these delays and developing interventions to address ethnic inequalities in secondary care.

## Method

### Design and databases

A population-based study was undertaken of patients in England with cancer using data from the Clinical Practice Research Datalink (CPRD Aurum) linked to Hospital Episode Statistics (HES) and the National Cancer Registration and Analysis Service (NCRAS) cancer registry. The scope and strengths of these databases are well documented.^21^^–^^24^ The CPRD is the world’s largest primary care database, with linkages to other health and area-based datasets used in this study.^21^^,^^22^ The CPRD Aurum (August 2019 release) used in this study includes anonymised primary care data from 890 practices in England, with over 28 million patients eligible for linkage to other healthcare databases. The dataset is representative of the English population in terms of geography, deprivation, age, gender, and ethnicity.^21^^,^^22^ HES contains detailed medical records of all admissions and outpatient appointments, including those at independent sector providers funded by the NHS.^23^ The NCRAS data contains records of all tumours diagnosed in England, including information on diagnosis date (pathological verification date), staging, treatment, patient-reported outcomes, and pathways to diagnosis.^24^

### Cancer sites and participants

Selected cancers were the first primary cancers diagnosed, including the four most common types (lung, breast, colorectal, and prostate) and three cancers more frequently diagnosed in Asian or Black ethnic groups (stomach, oesophageal, and myeloma), as well as ovarian cancer.^25^ Oesophagus and stomach cancer were merged into oesophagogastric cancer owing to their shared diagnostic features and suspected cancer referral criteria. Eligible patients were aged ≥40 years diagnosed via elective or urgent GP referral or 2-week wait (TWW) route between 2006 and 2016, using the latest NCRAS release at the start of the study. The TWW was an urgent referral route ensuring that referred patients were seen by a specialist within that time: it has since been renamed the urgent suspected cancer route.^26^

### Participants’ ethnicity

Patients’ ethnicity was identified from the CPRD or HES records as in previous studies.^27^^,^^28^ Ethnicity was defined using the UK’s census groupings, which includes the following: White (White British, White Irish, Any other White); Asian (Indian, Pakistani, Bangladeshi, Chinese, Other Asian); Black (Black Caribbean, Black African, other Black); mixed (White and Black Caribbean, White and Black African, White and Asian, Any other mixed); and Other ethnic group. For patients with multiple ethnicity codes, the authors of the current study assigned a single best ethnicity based on the most frequently recorded codes or in their absence, the most recently recorded code following established methods.^23^^,^^27^^,^^29^ Analysis focused on three broad categories (White, Asian, and Black) for which CPRD and HES data are highly complete and concordant. The mixed and other groups were excluded because of significant heterogeneity within the groups with poor concordance in their recording in the CPRD and HES.^27^

### Features of possible cancer

As in previous studies,^17^^,^^30^^,^^31^ suspected cancer features (Supplementary Table S1) were identified from the CPRD, based on the symptoms, signs, or blood test results in the original or revised National Institute for Health and Care Excellence (NICE) guidance.^16^^,^^32^ The index feature was defined as the first feature recorded in the year before diagnosis, as in previous studies.^17^^,^^30^

### Specialist referrals

Information on cancer specialist referral was available from the CPRD and HES. CPRD records of patients with cancer features were mapped to oncological specialties in HES (Supplementary Table S2), retaining only those with a recorded attendance date. It was not always possible to directly link referral request or appointment dates in HES to index feature presentations in CPRD, especially where the patient had multiple referral records. As a result, the referral date was defined as the earliest referral request date, and the specialist appointment date as the earliest appointment date, both recorded in HES. The final selection included only patients with a valid index feature date, referral request date, and date of attended specialist appointment.

### Defining DIs

Four distinct intervals within the diagnostic pathway were defined:

- the PI – the period between the index recorded feature and the earliest relevant referral request;
- the RI — the period between the earliest referral request and the earliest specialist appointment;
- the SI — the duration between the earliest specialist appointment and definitive diagnosis; and
- the total interval, usually labelled the DI.^18^ The DI is the period between the index recorded feature in primary care and the date of cancer diagnosis.

### Other variables

Information on patient age, sex (male/female), level of deprivation, and comorbidities were identified from the CPRD. As only the year of birth was available, a nominal birthday of 1 July was assigned to each patient. Individual-level deprivation was assessed using the quintiles of the Index of Multiple Deprivation (IMD), a composite measure of deprivation for small areas in England. The IMD is based on patients’ postcodes and covers various domains of material deprivation, including income, employment, education and skills, health, housing, crime rates, access to services, and the living environment.^33^ Comorbidities before cancer diagnosis were used to derive Cambridge Multimorbidity Score (CMS), based on the general-outcome weighting.^34^^,^^35^ The scores were analysed as quartiles of increasing morbidity burden.

### Statistical analysis

A logistic regression model was fitted to assess ethnic differences in the route to diagnosis (coded elective/urgent GP versus TWW), with adjustment for age, sex, deprivation, and CMS. Accelerated failure time (AFT) models investigated ethnic differences in DIs, adjusting for age, sex, deprivation, and CMS, with sandwich (‘robust’) standard errors to accommodate the lack of independence within practices. However, DIs vary greatly by cancer type, age, sex, deprivation, and the presence of comorbidities, all of which are also associated with ethnicity.^36^^–^^40^ Therefore, interactions were included between cancer and ethnicity, age, sex, deprivation, and CMS in the AFT models. Initial analyses demonstrated significant interactions between ethnicity and cancer type (*P* <0.001), suggesting that the association between ethnicity and each interval varies by site. To illustrate these differences, analyses of each interval is reported stratified by cancer. All analyses were carried out in Stata v18, with the STROBE framework for cohort studies guiding the reporting.^41^

## Results

Out of 142 437 patients with the seven selected cancers, 51 973 (36.5%) diagnosed via non-primary care routes were excluded (Supplementary Figure S1). Individuals with invalid date entries (*n* = 13 499) or lacking valid records of hospital attendance (*n* = 965), and those with records of secondary care attendance at a specialist unassociated with their cancer (*n* = 5029) were excluded. Table 1 shows the demographic characteristics of the 70 971 remaining patients.

**Table 1.** Characteristics of participants

**Characteristic**	**Asian**	**Black**	**White**	**All**
**Total participants *N* (%)**	1262 (1.8)	1592 (2.2)	68 117 (96.0)	70 971 (100)

**Sex, female**	669 (53.0)	482 (30.3)	29 511 (43.3)	30 662 (43.2)

**Age, years**				
Median (IQR)	65 (54–74)	66 (55–75)	71 (63–79)	71 (62–79)
40–49	230 (18.2)	249 (15.6)	5001 (7.34)	5480 (7.72)
50–59	258 (20.4)	347 (21.8)	8420 (12.4)	9025 (12.7)
60–69	312 (24.7)	363 (22.8)	18 217 (26.7)	18 892 (26.6)
70>	462 (36.6)	633 (39.8)	36 479 (53.6)	37 574 (52.9)

**IMD^a^**				
1 (least deprived)	200 (15.8)	88 (5.5)	17 219 (25.3)	17 507 (24.7)
2	228 (18.1)	99 (6.22)	15 968 (23.4)	16 295 (23.0)
3	280 (22.2)	252 (15.8)	13 692 (20.1)	14 224 (20.0)
4	257 (20.4)	451 (28.3)	11 377 (16.7)	12 085 (17.0)
5 (most deprived)	297 (23.5)	701 (44.1)	9839 (14.4)	10 837(15.3)

**Comorbidity score**				
0 (none)	97 (7.7)	118 (7.41)	5483 (8.05)	5698 (8.03)
1	259 (20.5)	362 (22.7)	14 468 (21.2)	15 089 (21.3)
2	298 (23.6)	367 (23.1)	13 889 (20.4)	14 554 (20.5)
3	303 (24.0)	386 (24.2)	16 328 (24.0)	17 017 (24.0)
4 (highest score)	305 (24.2)	359 (22.6)	17 949 (26.4)	18 613 (26.2)

**Site of cancer**				
Breast	459 (36.4)	327 (20.5)	16 529 (24.3)	17 315 (24.4)
Lung	132 (10.5)	105 (6.60)	11 567 (17.0)	11 804 (16.6)
Prostate	367 (29.1)	890 (55.9)	24 056 (35.3)	25 313 (35.7)
Colorectal	186 (14.7)	154 (9.7)	10 426 (15.3)	10 766 (15.2)
Oesophagogastric	43 (3.4)	47 (3.0)	2696 (4.0)	2786 (3.9)
Myeloma	35 (2.8)	57 (3.6)	1205 (1.8)	1297 (1.8)
Ovarian	40 (3.2)	12 (0.8)	1638 (2.4)	1690 (2.4)

*Data are n (%) unless otherwise indicated.*

a *IMD missing for 23 patients. IMD = Index of Multiple Deprivation. IQR = interquartile range.*

The majority of the 70 971 patients were of White ethnicity (68 117, 96.0%), with more males (40 309, 56.8%). At diagnosis, Asian and Black patients were younger, with a significant proportion resident in the most deprived areas. Comorbidity was similar across the three ethnic groups.

### Recorded index features of cancer

Breast cancer was dominated by breast lump (16 064/17 315, [92.8%]). The remaining sites were characterised by multiple features, with slight variations by ethnicity (Supplementary Information S1 and Supplementary Table S3).

### Ethnic differences in diagnostic routes

**Figure 1.**
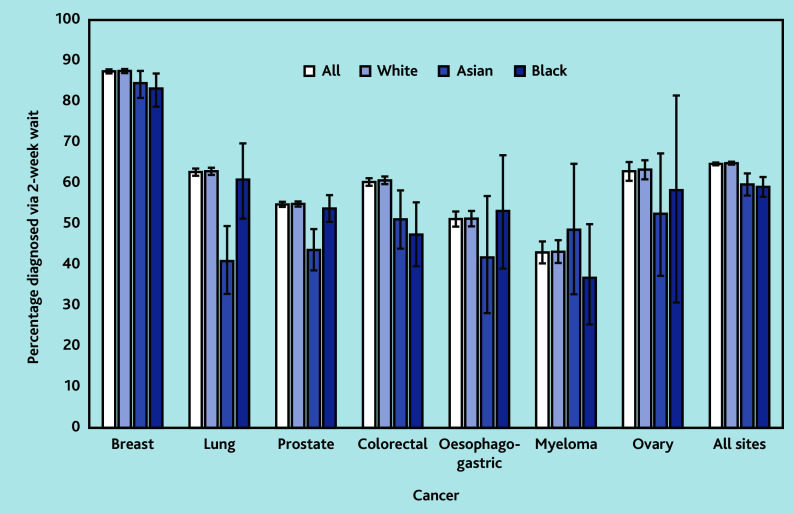
Proportion diagnosed via 2-week wait referral route. The errors lines are confidence intervals around the proportions. CI = 95%

Across all sites, of the 70 971 patients, White patients (44 240/68 117, 64.9%) were more often diagnosed via the TWW route than Black (941/1592, 59.1%) or Asian patients (753/1262, 59.7%) (Figure 1): adjusted odds ratio (AOR) 0.86 (95% CI = 0.78 to 0.96) for Black versus White patients and 0.67 (95% CI = 0.60 to 0.77) for Asian versus White patients.

The proportion diagnosed via TWW route was significantly lower for Black patients with breast (AOR 0.68, 95% CI = 0.51 to 0.92) or colorectal cancers (AOR 0.62, 95% CI = 0.45 to 0.86), and for Asian patients with lung (AOR 0.41, 95% CI = 0.29 to 0.58), prostate (AOR 0.64, 95% CI = 0.52 to 0.79), or colorectal cancers (AOR 0.73, 95% CI = 0.54 to 0.98) compared with White patients.

### Ethnic differences in DI

**Figure 2.**
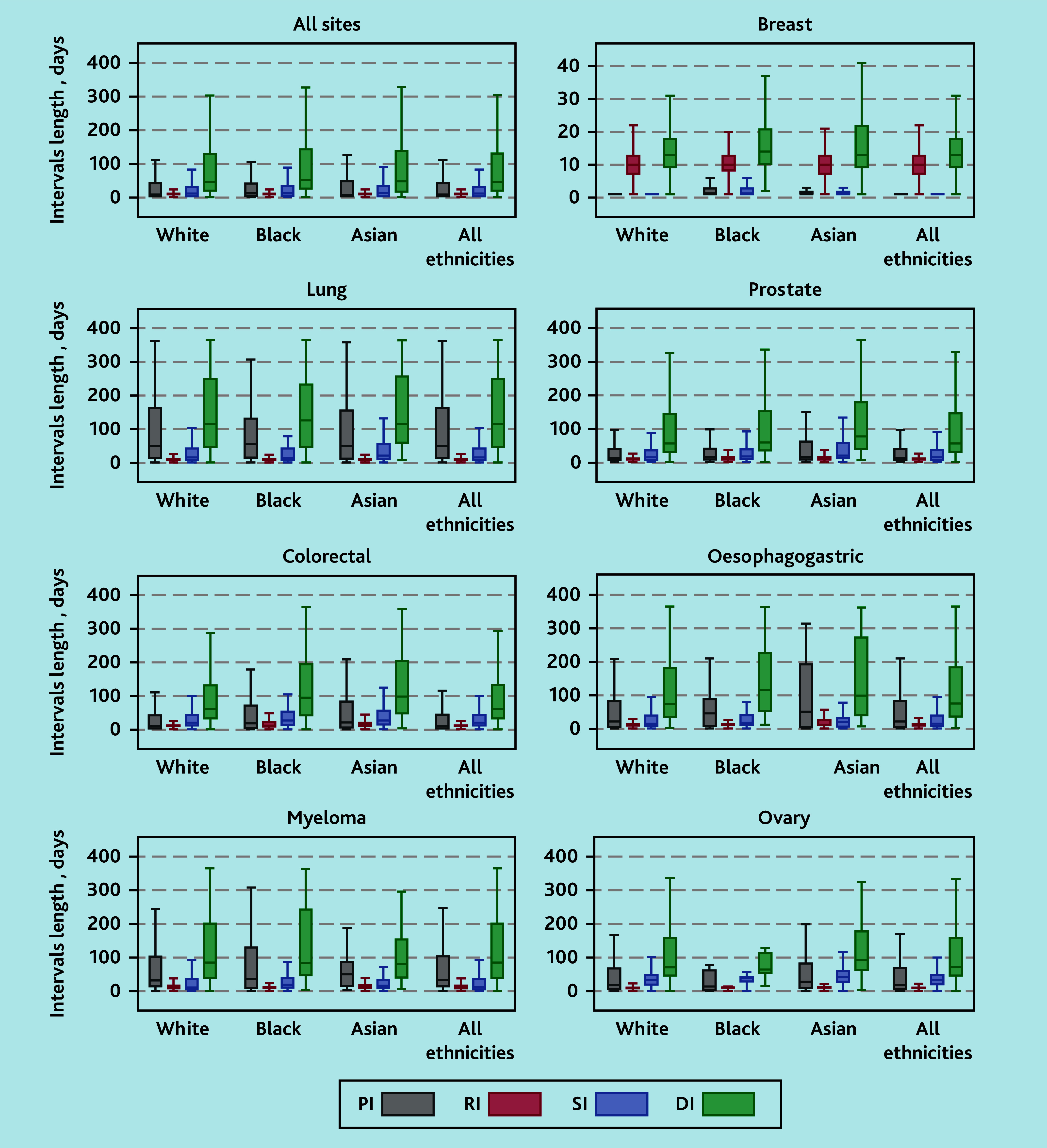
Boxplots of DIs (days) by cancer type and ethnicity. Each boxplot shows the median, quartiles, and ranges of DIs (in days), with outliers excluded for clarity. The corresponding values for the median, 25th percentile, 75th percentile, and 95th percentile are provided in Supplementary Table S4.

Several boxplots of DIs by site are illustrated in Figure 2 (additional details in Supplementary Table S4). Across the seven sites, the median DI was 46 days (IQR 18–133), with the longest DI observed in lung cancer (median 116 days, IQR 45–252) and the shortest in breast cancer (median 13, IQR 9–18 days). There were significant differences in DI by ethnicity.

**Figure 3.**
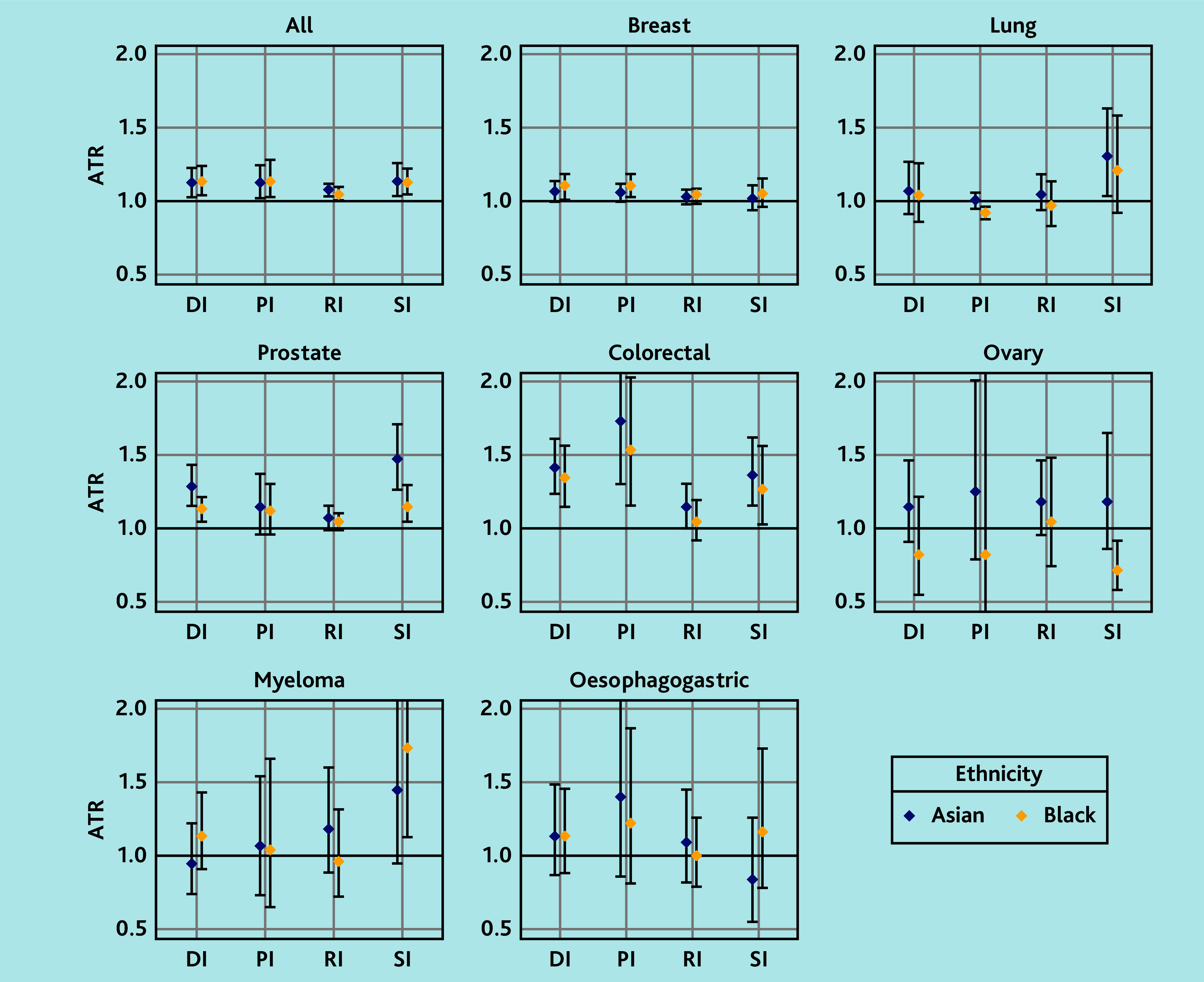
Association between ethnicity and DI by cancer site. Analysis was adjusted for age, sex, IMD, and CMS, with the White ethnic group as the reference group. ATR = adjusted time ratio. CMS = Cambridge Multimorbidity Score. DI = diagnostic interval. IMD = Index of Multiple Deprivation. PI = primary care interval. RI = referral interval. SI = secondary care interval.

Across all sites, the median DI was longer in Black (median 52 days, IQR 24–146) and Asian (median 48, IQR 15–141 days) than in White patients (median 46, IQR 18–133 days). After adjusting for age, sex, deprivation, and CMS, DI, was 14% longer among Black patients (adjusted time ratio [ATR] 1.14, 95% CI = 1.05 to 1.25) and 13% longer among Asian patients (ATR 1.13, 95% CI = 1.03 to 1.23) than White patients (Figure 3 and Supplementary Table S5).

In site-specific analyses, DIs were significantly longer in both Black and Asian patients compared with White patients for breast (Black: ATR 1.11, 95% CI = 1.03 to 1.19; Asian: ATR 1.07, 95% CI = 1.00 to 1.14), prostate (Black: ATR 1.13, 95% CI = 1.05 to 1.22; Asian: ATR 1.29, 95% CI = 1.16 to 1.44), and colorectal cancer (Black: ATR 1.35, 95% CI = 1.15 to 1.57; Asian: ATR 1.42, 95% CI = 1.24 to 1.62). DIs for lung, ovarian, myeloma, and oesophagogastric cancers showed little differences, although the sample size was substantially smaller for the last three sites.

### Ethnic differences in PI

The adjusted AFT models showed that across all sites, the PI was 15% longer in Black (ATR 1.15, 95% CI = 1.03 to 1.29) and 13% longer in Asian groups (ATR 1.13, 95% CI = 1.02 to 1.25) than in the White patient group.

Site-specific analyses revealed longer PIs for Black and Asian patients compared with White patients for breast cancer (Black: ATR 1.11, 95% CI = 1.03 to 1.19; Asian: ATR 1.06, 95% CI = 1.00 to 1.12) and colorectal cancer (Black: ATR 1.54, 95% CI = 1.16 to 2.04; Asian: ATR 1.74, 95% CI = 1.31 to 2.32), but a shorter for Black patients with lung cancer (ATR 0.92, 95% CI = 0.88 to 0.96) compared with White patients. There was no evidence of ethnic differences in the PI for ovarian, myeloma, prostate, and oesophagogastric cancers.

### Ethnic differences in RI

After adjusting for covariates, RIs across all sites was found to be 5% longer among Black patients (ATR ratio 1.05, 95% CI = 1.01 to 1.10) and 8% longer among Asian patients (ATR ratio 1.08, 95% CI = 1.04 to 1.12) compared with White patients. Site-specific analyses revealed little evidence of ethnic differences in RI except for colorectal cancer, for which the Asian group had a longer interval than the White group (ATR ratio 1.15, 95% CI = 1.00 to 1.31).

### Ethnic differences in SI

After adjusting for covariates, the SI across all sites was found to be 13% longer among Black patients (ATR ratio 1.13, 95% CI = 1.05 to 1.23) and 14% longer among Asian patients (ATR ratio 1.14, 95% CI = 1.04 to 1.26) than White patients.

Site-specific analyses revealed a longer SI among Black patients with prostate cancer (ATR ratio 1.16, 95% CI = 1.04 to 1.30), colorectal cancer (ART ratio 1.27, 95% CI = 1.03 to 1.57), and myeloma (ATR ratio 1.74, 95% CI = 1.13 to 2.67) than White patients. Similarly, a longer SI was found among Asian patients with lung (ATR ratio 1.31, 95% CI = 1.05 to 1.64), prostate (ATR ratio 1.48, 95% CI = 1.27 to 1.72), and colorectal cancer (ATR ratio 1.37, 95% CI = 1.16 to 1.63) compared with White patients. However, Black patients with ovarian cancer had a shorter SI than White patients (ATR ratio 0.72, 95% CI = 0.58 to 0.92).

## Discussion

### Summary

Unexpectedly, evidence was found of ethnic inequalities across all three intervals for six of the seven cancers, not just the PI. This is in the context of an average DI of nearly 4 months for lung cancer and >2 months for colorectal, myeloma, ovarian, and oesophagogastric cancers across the entire population, bearing in mind that a delay of ≥2 months is associated with poorer cancer survival rates.^42^

### Strengths and limitations

The cohort consists of patients diagnosed between 2006 and 2016, based on the most recent NCRAS data available at the start of the study, and so omits any effects from COVID-19. ‘Combined ethnic categories’ were used for simplicity, recognising that this approach conceals heterogeneity within ethnic subgroups. The current analysis was restricted to patients who had a recorded cancer feature in primary care, were GP-referred, and had relevant referral, appointment, and specialist attendance dates in HES. Although these restrictions may have introduced bias by excluding patients presenting elsewhere or with non-NICE qualifying symptoms, they ensure that the results are the most accurate estimates of DI involving primary care. Those excluded were broadly similar to those included (results not shown); therefore, the impact of this restriction is likely to be minimal. Patients from the mixed and other ethnic groups were excluded owing to significant heterogeneity within these groups and poor concordance in their recording in the CPRD and HES.^27^ The other group, in particular, includes patients listed as of ‘unknown ethnicity’ in HES or those labelled with an ethnicity not categorised as Asian, Black, mixed, or White ethnic groups in the census or CPRD. Both groups make up <4% of the entire cohort, so their exclusion has minimal impact on the current sample. Importantly, in the same author group’s previous work, the authors were unable to interpret the findings from these groups, making their inclusion in the present study less valuable.^17^ Finally, a limitation is that the current analysis for some cancers, particularly oesophagogastric cancer, ovarian cancer, and myeloma, had small sample sizes perhaps providing insufficient power to see differences.

### Comparison with existing literature

The median DI for all patients in this study was 48 days, shorter than the 55 days reported in the same author group’s previous study, which included DI estimates for patients with recorded symptoms in primary care, regardless of whether they were diagnosed via primary care or hospital routes.^17^ As highlighted above, the current study provides a more precise DI estimate by focusing exclusively on patients diagnosed via primary care routes. The current findings of ethnic differences in DI across all sites, as well as for patients with breast, prostate, and colorectal cancers align with those reported in earlier studies.^17^^,^^20^ Similarly, the current findings of ethnic differences in PI for breast cancer, RI for colorectal cancer, and SI for lung cancer are consistent with results from a previous national survey of NHS patients.^20^ However, the current study found evidence of ethnic differences in the PI for lung and colorectal cancers, as well as the SI for myeloma, prostate, colorectal, and ovarian cancers, contrasting with those reported in that survey.^20^

### Implications for research and practice

For breast cancer, the main ethnic difference lay in the PI, which was slightly longer for Asian and Black patients. This may, in part, reflect the symptoms reported during consultations, resulting in Black women being less often diagnosed via the TWW route. Although a breast lump is the commonest feature of breast cancer,^30^^,^^43^ Black patients had the highest proportion with breast pain, a low-risk symptom of cancer,^44^ which is unlikely to trigger an expedited diagnosis. To minimise inequalities in the PI, GPs should carefully evaluate Black women with breast pain and other non-lump features and consider expedited referral where appropriate. However, further studies are necessary to assess the predictive value of non-lump symptoms, particularly in Black women.

For prostate cancer, the study found little evidence of ethnic differences in the PI and RI. However, Asian and Black patients had a longer SI compared with White patients, with medians of 21 and 19 days versus 16 days for White patients. This was unexpected, considering that the risk of cancer (and associated psychological distress) rises sharply once referred to secondary care.^45^^,^^46^ Although these differences are small, larger differences occur in the tail of the distribution, with the 95th percentile of the SI being over 40 days longer in Asian patients than White patients. The absence of evidence of ethnic differences in the PI is counterintuitive but may suggest equal access to primary care assessment for suspected prostate cancer. As there is little evidence of ethnic differences in pre-presentational delay among symptomatic men,^47^^–^^49^ this finding implies that ethnic inequalities in prostate cancer outcomes^50^ are likely because of the disease biology rather than inequities in the diagnostic pathways.

Lung cancer patients had the longest DI, driven largely by prolonged PIs and SIs. The median DI was 116 days, similar to 117 days previously reported in a study where DI estimates included pre-presentation interval.^51^ As previously reported,^17^^,^^20^ the current study found no evidence of ethnic differences in DI and RI, but Black patients had a shorter PI than White patients. One explanation could be late-stage presentation: Black patients, who are less likely to smoke^52^ and have a lower incidence of lung cancer,^25^ are more likely to present with advanced-stage disease.^1^ They might appear more obviously ill at presentation, thereby prompting a fast-track referral to secondary care, although Black patients with lung cancer (in the current study) were equally likely as White patients to be referred via the TWW route. A critical finding was that Asian patients had a longer SI compared with White patients. The reason for this disparity is unclear, although Asian patients — compared with White patients — were less likely to be referred via the TWW route despite both presenting with similar lung features.^43^ Further studies are necessary to understand the underlying causes and develop strategies to address ethnic disparities in secondary care.

For colorectal cancer, longer PIs and SIs were found among Asian and Black patients, and longer RIs among Asian patients compared with White patients. It is possible that the differences in PIs are associated with differential acceptance of primary care investigations offered, although this aspect has not been specifically studied. Before the Faecal Immunochemical Test (FIT) was introduced in 2017, GPs used the faecal occult blood test and digital rectal examination to assess patients with suspected colorectal cancer — both of which some patients may have found uncomfortable.^53^^,^^54^ The FIT test is considered ‘much less disgusting’ in screening settings.^55^ It is unclear whether this is the case among patients presenting with symptoms in primary care making this a potential area for future work.

The authors are uncertain why RIs (in Asian patients) and SIs were longer in Asian and Black patients with colorectal cancer. Neither can the authors explain the observed longer SI among Black patients with myeloma. The findings of shorter SIs among Black patients with ovarian cancer must be interpreted with caution considering the relatively small sample of Black patients. Further studies should examine the causes of disparities in RIs and SIs, particularly for myeloma and colorectal cancer considering that incidence is higher particularly, in the Black group.

In conclusion, the study found evidence of ethnic disparities in DIs, including the PI and SI, in six of the seven cancer types studied. Although modest overall, these differences are not expected in a universally accessible healthcare system. They point to major shortcomings in the organisation and delivery of health care in the UK, unfavourable to ethnic minorities. Further studies are necessary to fully understand the mechanisms contributing to these disparities and to develop interventions to address them.

## Data

This study used CPRD-linked data, access to which is subject to protocol approval by an Independent Scientific Advisory Committee, and under which conditions data cannot be shared directly.
